# Dibromido(2,9-dimethyl-1,10-phenanthroline-κ^2^ *N*,*N*′)cadmium

**DOI:** 10.1107/S1600536811050069

**Published:** 2011-11-30

**Authors:** Ismail Warad, Ahmed Boshaala, Saud I. Al-Resayes, Salem S. Al-Deyab, Mohamed Rzaigui

**Affiliations:** a Department of Chemistry, King Saud University, PO Box 2455, Riyadh 11451, Saudi Arabia; b Department of Chemistry, AN-Najah National University, PO Box 7, Nablus, Palestinian Territories; c Petrochemical Research Chair, College of Science, King Saud University, Riyadh, Saudi Arabia; d Laboratoire de Chimie des Matériaux, Faculté des Sciences de Bizerte, 7021 Zarzouna Bizerte, Tunisia

## Abstract

In the title complex, [CdBr_2_(C_14_H_12_N_2_)], the Cd^II^ ion is tetra­coordinated by two N atoms of the bidentate 2,9-dimethyl-1,10-phenanthroline ligand and two bromide ions in a substanti­ally distorted CdN_2_Br_2_ tetra­hedral geometry. In the crystal, inversion dimers linked by pairs of weak C—H⋯Br bonds generate *R*_2_^2^(14) loops. Aromatic π–π stacking [shortest centroid–centroid separation = 3.633 (2) Å] inter­actions occur within, and also link, the dimers into chains propagating parallel to [100].

## Related literature

For related structures, see: Preston & Kennard (1969 ▶); Lange *et al.* (2000 ▶); Alizadeh *et al.* (2009 ▶); Wang & Zhong (2009 ▶); Warad *et al.* (2011 ▶). For background to π–π stacking inter­actions, see: Janiak (2000 ▶). 
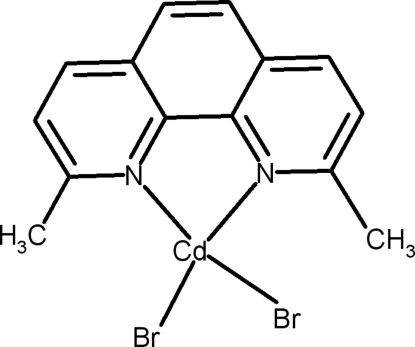


## Experimental

### 

#### Crystal data

[CdBr_2_(C_14_H_12_N_2_)]
*M* *_r_* = 480.48
Monoclinic, 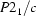

*a* = 7.889 (4) Å
*b* = 10.519 (3) Å
*c* = 18.712 (2) Å
β = 97.69 (3)°
*V* = 1538.8 (9) Å^3^
*Z* = 4
Ag *K*α radiation
λ = 0.56087 Å
μ = 3.53 mm^−1^
*T* = 293 K
0.30 × 0.25 × 0.17 mm

#### Data collection

Enraf–Nonius CAD-4 diffractometer
Absorption correction: multi-scan (*SORTAV*; Blessing, 1995 ▶) *T*_min_ = 0.469, *T*_max_ = 0.534
9813 measured reflections
7516 independent reflections
2986 reflections with *I* > 2σ(*I*)
*R*_int_ = 0.029
2 standard reflections every 120 min  intensity decay: 1%

#### Refinement

*R*[*F* ^2^ > 2σ(*F* ^2^)] = 0.063
*wR*(*F* ^2^) = 0.177
*S* = 0.98
7516 reflections
174 parameters
H-atom parameters constrained
Δρ_max_ = 1.41 e Å^−3^
Δρ_min_ = −0.93 e Å^−3^

### 

Data collection: *CAD-4 EXPRESS* (Enraf–Nonius, 1994 ▶); cell refinement: *CAD-4 EXPRESS*; data reduction: *XCAD4* (Harms & Wocadlo, 1995 ▶); program(s) used to solve structure: *SHELXS86* (Sheldrick, 2008 ▶); program(s) used to refine structure: *SHELXL97* (Sheldrick, 2008 ▶); molecular graphics: *ORTEPIII* (Burnett & Johnson, 1996 ▶) and *DIAMOND* (Brandenburg & Putz, 2005 ▶); software used to prepare material for publication: *WinGX* (Farrugia, 1999 ▶).

## Supplementary Material

Crystal structure: contains datablock(s) I, global. DOI: 10.1107/S1600536811050069/hb6522sup1.cif

Structure factors: contains datablock(s) I. DOI: 10.1107/S1600536811050069/hb6522Isup2.hkl

Additional supplementary materials:  crystallographic information; 3D view; checkCIF report

## Figures and Tables

**Table:** 

Cd1—N2	2.273 (4)
Cd1—N1	2.294 (4)
Cd1—Br2	2.5050 (10)
Cd1—Br1	2.5120 (13)

**Table:** 

N2—Cd1—N1	73.81 (14)

**Table 2:** Hydrogen-bond geometry (Å, °)

*D*—H⋯*A*	*D*—H	H⋯*A*	*D*⋯*A*	*D*—H⋯*A*
C5—H5⋯Br1^i^	0.93	2.98	3.805 (5)	149

Symmetry code: (i) 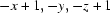.

**Fig. 1.**
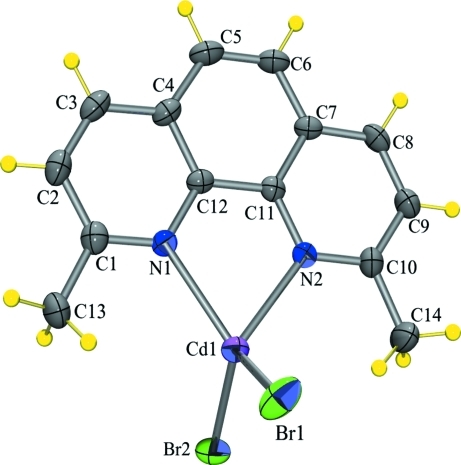
An ORTEP (Burnett & Johnson, 1996) view of (I). Displacement ellipsoids are drawn at the 30% probability level. H atoms are represented as small spheres of arbitrary radii.

**Fig. 2.**
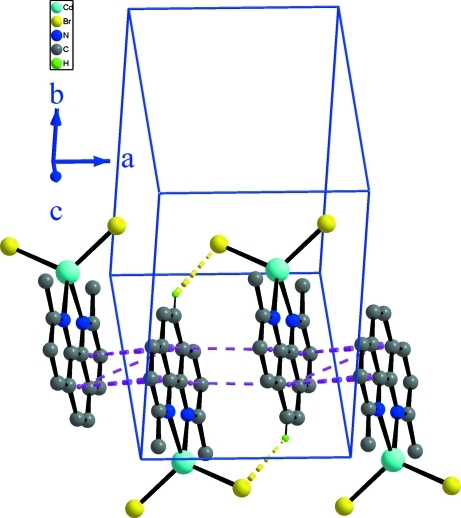
A view of the [CdBr2(dmphen)2]n chains extending along a axis. The H-atoms not involved in H-bonding have been omitted.

## supplementary crystallographic information

### Comment

The reaction of cadmium(II) halides, CdX_2_, with nitrogen-based ligands (*L*) yields mixed-ligand complexes. The number of ligands bound to the metal cation is influenced greatly by both the chemical nature and geometry of ligand *L* and the type of halogen *X* (Lange *et al.*, 2000). In this sense, we report herein synthesis and crystal structure of a new Cd^II^ complex, [CdBr_2_(dmphen)] (I), where dmphen = 2,9-dimethyl-1,10-phenanthroline.

The molecular structure of (I), along with the numbering scheme, is shown in Fig. 1. The Cd^II^ cation is located on a general position in a tetrahedral environment built up from two nitrogen atoms (N1, N2) of one dmphen bidentate ligand and two bromide ions (Br1, Br2). Similar coordination geometry around the central atom has been observed in other metal complexes involving the same ligand (dmphen) such as [HgBr_2_(dmphen)], (Alizadeh *et al.*, 2009), [ZnCl_2_(dmphen)] (Preston *et al.*, 1969), [CuCl_2_(dmphen)] (Wang *et al.*, 2009).

Geometrical analysis of the bond lengths and angles around the cadmium atom shows that the CdBr_2_N_2_ tetrahedron, where the Cd shift from the gravity center is δ = 0.249 Å, is less distorted that the CdI_2_N_2_ (δ = 0.356 Å) in the [CdI_2_(dmphen)] structure (Warad *et al.*, 2011). This can be explaned by the large size and π-acid character of the iodine atom.

It should be noted also that, in the crystal packing of [CdI_2_(dmphen)], there is no C—H···I H-bond, while in [CdBr_2_(dmphen)], weak intermolecular C—H···Br bonds (2.98 (3) Å) link the complex molecules into dimeric clusters. Additional π–π aromatic stacking interactions, between the dmphen rings of neighboring molecules, associate these clusters into chains parallel to the *a* axis (Fig. 2). The π–π contacts involve the dmphen rings N1C1C2C3C4C12 (centroid *Cg*1), C4C5C6C7C11C12 (centroid *Cg*2) and N2C10C9C8C7C11 (centroid *Cg*3) between which exist the centroid-centroid distances *Cg*1···*Cg*3^i^ (3.634 Å)) [symmetry code: (i) 1 - *x*, -*y*, 1 - *z*], *Cg*2···*Cg*3^ii^ (3.722 Å) and *Cg*3···*Cg*3^ii^ (3.705 Å) [symmetry code: (ii) 2 - *x*, -*y*, 1 - *z*], which are less than the maximum value (3.8 Å) regarded as relevant for π–π interactions (Janiak, 2000).

### Experimental

This complex was prepared by a procedure similar to that used for [CdI_2_(dmphen)] (Warad *et al.*, 2011). A mixture of cadimium bromide (CdBr_2_.4H_2_O, 82.7 mg, 0.24 mmol) in methanol (20 ml) and dmphen (50.0 mg, 0.24 mmol) in dichloromethane (10 ml) is stirred for 2H at room temperature. The obtained solution was concentrated to about 2 ml under reduced pressure and mixed to 40 ml of n-hexane. This causes the precipitation of white powder which was filtered, dried and used for the preparation of colourless prisms of (I) by slow diffusion of diethyl ether into a solution of the complex in dichloromethane.

### Refinement

All H atoms attached to C atoms were fixed geometrically and treated as riding, with C—H = 0.93 Å and 0.96 Å and with *U*_iso_(H) = 1.2Ueq(C) and *U*_iso_(H) = 1.5Ueq(C_methyl_).

### Crystal data

**Table:**

[CdBr_2_(C_14_H_12_N_2_)]	*F*(000) = 912
*M**_r_* = 480.48	*D*_x_ = 2.074 Mg m^−^^3^
Monoclinic, *P*2_1_/*c*	Ag *K*α radiation, λ = 0.56087 Å
*a* = 7.889 (4) Å	Cell parameters from 25 reflections
*b* = 10.519 (3) Å	θ = 9–11°
*c* = 18.712 (2) Å	µ = 3.53 mm^−^^1^
β = 97.69 (3)°	*T* = 293 K
*V* = 1538.8 (9) Å^3^	Prism, colorless
*Z* = 4	0.30 × 0.25 × 0.17 mm

### Data collection

**Table:**

Enraf–Nonius CAD-4 diffractometer	2986 reflections with *I* > 2σ(*I*)
Radiation source: fine-focus sealed tube	*R*_int_ = 0.029
graphite	θ_max_ = 28.0°, θ_min_ = 2.1°
non–profiled ω scans	*h* = −13→13
Absorption correction: multi-scan (*SORTAV*; Blessing, 1995)	*k* = −2→17
*T*_min_ = 0.469, *T*_max_ = 0.534	*l* = −2→31
9813 measured reflections	2 standard reflections every 120 min
7516 independent reflections	intensity decay: 1%

### Refinement

**Table:**

Refinement on *F*^2^	Primary atom site location: structure-invariant direct methods
Least-squares matrix: full	Secondary atom site location: difference Fourier map
*R*[*F*^2^ > 2σ(*F*^2^)] = 0.063	Hydrogen site location: inferred from neighbouring sites
*wR*(*F*^2^) = 0.177	H-atom parameters constrained
*S* = 0.98	*w* = 1/[σ^2^(*F*_o_^2^) + (0.0769*P*)^2^] where *P* = (*F*_o_^2^ + 2*F*_c_^2^)/3
7516 reflections	(Δ/σ)_max_ = 0.001
174 parameters	Δρ_max_ = 1.41 e Å^−^^3^
0 restraints	Δρ_min_ = −0.93 e Å^−^^3^

### Special details

**Table:**

Geometry. All e.s.d.'s (except the e.s.d. in the dihedral angle between two l.s. planes) are estimated using the full covariance matrix. The cell e.s.d.'s are taken into account individually in the estimation of e.s.d.'s in distances, angles and torsion angles; correlations between e.s.d.'s in cell parameters are only used when they are defined by crystal symmetry. An approximate (isotropic) treatment of cell e.s.d.'s is used for estimating e.s.d.'s involving l.s. planes.
Refinement. Refinement of *F*^2^ against ALL reflections. The weighted *R*-factor *wR* and goodness of fit *S* are based on *F*^2^, conventional *R*-factors *R* are based on *F*, with *F* set to zero for negative *F*^2^. The threshold expression of *F*^2^ > σ(*F*^2^) is used only for calculating *R*-factors(gt) *etc*. and is not relevant to the choice of reflections for refinement. *R*-factors based on *F*^2^ are statistically about twice as large as those based on *F*, and *R*- factors based on ALL data will be even larger.

### Fractional atomic coordinates and isotropic or equivalent isotropic displacement parameters (Å^2^)

**Table:**

	*x*	*y*	*z*	*U*_iso_*/*U*_eq_	
Cd1	0.71490 (5)	0.28442 (4)	0.39225 (2)	0.05032 (14)	
Br1	0.44548 (9)	0.32557 (8)	0.30765 (4)	0.0833 (2)	
Br2	0.93295 (10)	0.45768 (6)	0.39288 (4)	0.0766 (2)	
N1	0.6886 (5)	0.1781 (4)	0.4972 (2)	0.0422 (8)	
N2	0.8075 (5)	0.0829 (4)	0.3769 (2)	0.0409 (8)	
C1	0.6270 (6)	0.2257 (5)	0.5546 (3)	0.0501 (11)	
C2	0.6113 (7)	0.1479 (6)	0.6149 (3)	0.0561 (13)	
H2	0.5692	0.1819	0.6549	0.067*	
C3	0.6578 (7)	0.0234 (6)	0.6146 (3)	0.0553 (14)	
H3	0.6462	−0.0278	0.6541	0.066*	
C4	0.7229 (6)	−0.0278 (5)	0.5551 (3)	0.0462 (11)	
C5	0.7760 (6)	−0.1572 (5)	0.5505 (3)	0.0562 (13)	
H5	0.7680	−0.2113	0.5892	0.067*	
C6	0.8373 (7)	−0.2031 (5)	0.4918 (3)	0.0530 (12)	
H6	0.8724	−0.2875	0.4910	0.064*	
C7	0.8489 (6)	−0.1248 (4)	0.4314 (3)	0.0412 (10)	
C8	0.9065 (6)	−0.1674 (5)	0.3680 (3)	0.0511 (12)	
H8	0.9394	−0.2518	0.3644	0.061*	
C9	0.9152 (7)	−0.0873 (5)	0.3119 (3)	0.0535 (12)	
H9	0.9543	−0.1166	0.2702	0.064*	
C10	0.8653 (7)	0.0387 (5)	0.3170 (3)	0.0518 (12)	
C11	0.7981 (5)	0.0045 (4)	0.4331 (2)	0.0375 (9)	
C12	0.7359 (5)	0.0532 (4)	0.4963 (2)	0.0389 (10)	
C13	0.5798 (8)	0.3629 (6)	0.5518 (3)	0.0663 (16)	
H13A	0.6777	0.4129	0.5704	0.100*	
H13B	0.4894	0.3773	0.5805	0.100*	
H13C	0.5421	0.3870	0.5028	0.100*	
C14	0.8682 (10)	0.1297 (6)	0.2557 (3)	0.079 (2)	
H14A	0.7966	0.2013	0.2624	0.119*	
H14B	0.8266	0.0879	0.2112	0.119*	
H14C	0.9833	0.1582	0.2543	0.119*	

### Atomic displacement parameters (Å^2^)

**Table:**

	*U*^11^	*U*^22^	*U*^33^	*U*^12^	*U*^13^	*U*^23^
Cd1	0.0656 (3)	0.03953 (19)	0.0473 (2)	0.00785 (18)	0.01316 (17)	0.00728 (16)
Br1	0.0686 (4)	0.1170 (6)	0.0645 (4)	0.0160 (4)	0.0094 (3)	0.0320 (4)
Br2	0.1037 (5)	0.0481 (3)	0.0791 (4)	−0.0126 (3)	0.0164 (4)	0.0111 (3)
N1	0.047 (2)	0.042 (2)	0.0384 (19)	−0.0033 (17)	0.0057 (16)	0.0018 (16)
N2	0.045 (2)	0.0379 (19)	0.0400 (19)	0.0016 (16)	0.0067 (17)	0.0003 (16)
C1	0.041 (2)	0.058 (3)	0.052 (3)	−0.002 (2)	0.009 (2)	−0.005 (3)
C2	0.050 (3)	0.076 (4)	0.044 (3)	−0.004 (3)	0.013 (2)	−0.006 (3)
C3	0.049 (3)	0.076 (4)	0.042 (3)	−0.010 (3)	0.009 (2)	0.012 (3)
C4	0.039 (2)	0.056 (3)	0.043 (2)	−0.005 (2)	0.006 (2)	0.011 (2)
C5	0.054 (3)	0.052 (3)	0.061 (3)	−0.003 (3)	0.004 (3)	0.021 (3)
C6	0.054 (3)	0.039 (2)	0.066 (3)	0.000 (2)	0.005 (3)	0.013 (2)
C7	0.036 (2)	0.037 (2)	0.050 (3)	−0.0016 (18)	0.003 (2)	0.006 (2)
C8	0.051 (3)	0.038 (2)	0.064 (3)	0.007 (2)	0.007 (2)	−0.009 (2)
C9	0.062 (3)	0.047 (3)	0.053 (3)	0.003 (2)	0.011 (2)	−0.008 (2)
C10	0.063 (3)	0.051 (3)	0.042 (2)	0.000 (2)	0.010 (2)	−0.006 (2)
C11	0.033 (2)	0.038 (2)	0.040 (2)	−0.0021 (17)	−0.0001 (18)	−0.0001 (18)
C12	0.034 (2)	0.039 (2)	0.042 (2)	−0.0026 (18)	−0.0004 (18)	0.0068 (19)
C13	0.082 (4)	0.057 (3)	0.065 (4)	0.008 (3)	0.026 (3)	−0.010 (3)
C14	0.129 (6)	0.060 (4)	0.057 (3)	0.013 (4)	0.041 (4)	0.006 (3)

### Geometric parameters (Å, °)

**Table:**

Cd1—N2	2.273 (4)	C5—H5	0.9300
Cd1—N1	2.294 (4)	C6—C7	1.412 (7)
Cd1—Br2	2.5050 (10)	C6—H6	0.9300
Cd1—Br1	2.5120 (13)	C7—C8	1.399 (7)
N1—C1	1.334 (6)	C7—C11	1.419 (6)
N1—C12	1.367 (6)	C8—C9	1.355 (8)
N2—C11	1.347 (6)	C8—H8	0.9300
N2—C10	1.348 (6)	C9—C10	1.389 (7)
C1—C2	1.412 (8)	C9—H9	0.9300
C1—C13	1.489 (8)	C10—C14	1.496 (8)
C2—C3	1.360 (8)	C11—C12	1.433 (6)
C2—H2	0.9300	C13—H13A	0.9600
C3—C4	1.395 (7)	C13—H13B	0.9600
C3—H3	0.9300	C13—H13C	0.9600
C4—C12	1.405 (6)	C14—H14A	0.9600
C4—C5	1.430 (8)	C14—H14B	0.9600
C5—C6	1.347 (8)	C14—H14C	0.9600
			
N2—Cd1—N1	73.81 (14)	C8—C7—C6	123.7 (4)
N2—Cd1—Br2	116.56 (10)	C8—C7—C11	116.8 (4)
N1—Cd1—Br2	119.43 (10)	C6—C7—C11	119.5 (5)
N2—Cd1—Br1	109.88 (10)	C9—C8—C7	120.9 (5)
N1—Cd1—Br1	117.25 (10)	C9—C8—H8	119.6
Br2—Cd1—Br1	113.59 (4)	C7—C8—H8	119.6
C1—N1—C12	120.1 (4)	C8—C9—C10	119.7 (5)
C1—N1—Cd1	126.2 (3)	C8—C9—H9	120.1
C12—N1—Cd1	113.6 (3)	C10—C9—H9	120.1
C11—N2—C10	119.9 (4)	N2—C10—C9	121.1 (5)
C11—N2—Cd1	114.8 (3)	N2—C10—C14	117.3 (5)
C10—N2—Cd1	125.2 (3)	C9—C10—C14	121.6 (5)
N1—C1—C2	120.4 (5)	N2—C11—C7	121.6 (4)
N1—C1—C13	116.8 (5)	N2—C11—C12	119.1 (4)
C2—C1—C13	122.8 (5)	C7—C11—C12	119.4 (4)
C3—C2—C1	120.1 (5)	N1—C12—C4	121.5 (4)
C3—C2—H2	119.9	N1—C12—C11	118.6 (4)
C1—C2—H2	119.9	C4—C12—C11	119.8 (4)
C2—C3—C4	120.2 (5)	C1—C13—H13A	109.5
C2—C3—H3	119.9	C1—C13—H13B	109.5
C4—C3—H3	119.9	H13A—C13—H13B	109.5
C3—C4—C12	117.7 (5)	C1—C13—H13C	109.5
C3—C4—C5	123.9 (5)	H13A—C13—H13C	109.5
C12—C4—C5	118.5 (5)	H13B—C13—H13C	109.5
C6—C5—C4	122.0 (5)	C10—C14—H14A	109.5
C6—C5—H5	119.0	C10—C14—H14B	109.5
C4—C5—H5	119.0	H14A—C14—H14B	109.5
C5—C6—C7	120.8 (5)	C10—C14—H14C	109.5
C5—C6—H6	119.6	H14A—C14—H14C	109.5
C7—C6—H6	119.6	H14B—C14—H14C	109.5
			
N2—Cd1—N1—C1	−178.5 (4)	C7—C8—C9—C10	−0.4 (8)
Br2—Cd1—N1—C1	69.7 (4)	C11—N2—C10—C9	0.3 (8)
Br1—Cd1—N1—C1	−74.1 (4)	Cd1—N2—C10—C9	−178.3 (4)
N2—Cd1—N1—C12	−1.4 (3)	C11—N2—C10—C14	178.4 (5)
Br2—Cd1—N1—C12	−113.2 (3)	Cd1—N2—C10—C14	−0.2 (7)
Br1—Cd1—N1—C12	103.0 (3)	C8—C9—C10—N2	−0.3 (8)
N1—Cd1—N2—C11	1.3 (3)	C8—C9—C10—C14	−178.4 (6)
Br2—Cd1—N2—C11	116.6 (3)	C10—N2—C11—C7	0.4 (7)
Br1—Cd1—N2—C11	−112.4 (3)	Cd1—N2—C11—C7	179.1 (3)
N1—Cd1—N2—C10	180.0 (4)	C10—N2—C11—C12	−179.9 (4)
Br2—Cd1—N2—C10	−64.7 (4)	Cd1—N2—C11—C12	−1.1 (5)
Br1—Cd1—N2—C10	66.3 (4)	C8—C7—C11—N2	−1.0 (6)
C12—N1—C1—C2	0.5 (7)	C6—C7—C11—N2	179.8 (4)
Cd1—N1—C1—C2	177.4 (4)	C8—C7—C11—C12	179.2 (4)
C12—N1—C1—C13	179.4 (5)	C6—C7—C11—C12	0.0 (6)
Cd1—N1—C1—C13	−3.6 (6)	C1—N1—C12—C4	−0.6 (7)
N1—C1—C2—C3	−0.5 (8)	Cd1—N1—C12—C4	−177.8 (3)
C13—C1—C2—C3	−179.5 (5)	C1—N1—C12—C11	178.7 (4)
C1—C2—C3—C4	0.7 (8)	Cd1—N1—C12—C11	1.4 (5)
C2—C3—C4—C12	−0.8 (7)	C3—C4—C12—N1	0.7 (7)
C2—C3—C4—C5	179.5 (5)	C5—C4—C12—N1	−179.5 (4)
C3—C4—C5—C6	179.6 (5)	C3—C4—C12—C11	−178.5 (4)
C12—C4—C5—C6	−0.1 (8)	C5—C4—C12—C11	1.2 (7)
C4—C5—C6—C7	−1.1 (8)	N2—C11—C12—N1	−0.2 (6)
C5—C6—C7—C8	−178.1 (5)	C7—C11—C12—N1	179.6 (4)
C5—C6—C7—C11	1.1 (7)	N2—C11—C12—C4	179.0 (4)
C6—C7—C8—C9	−179.8 (5)	C7—C11—C12—C4	−1.2 (6)
C11—C7—C8—C9	1.0 (7)		

### Hydrogen-bond geometry (Å, °)

**Table:**

*D*—H···*A*	*D*—H	H···*A*	*D*···*A*	*D*—H···*A*
C5—H5···Br1^i^	0.93	2.98	3.805 (5)	149

Symmetry codes: (i) −*x*+1, −*y*, −*z*+1.
